# Supplementary material for: How the motor aspect of speaking influences the blink rate
Source: PLoS One. 2021 Oct 8;16(10):e0258322. doi: 10.1371/journal.pone.0258322 (PMC8500445; doi:10.1371/journal.pone.0258322)
Supplement: Attachment — Submitted filename: answer_to_reviewers_editor.docx [file pone.0258322.s001.docx]

Editor

A primary concern of Reviewer 1 is that we cannot be sure whether participants engaged in “talking inside the head”. I share this concern, but feel that it extends to several other conditions: “talking without sound” and “listen to someone else”. None of these tasks included a reliability measure. For example, you asked your participants to listen to someone else, but did they? Simple reliability check such as “you’ll be asked a question about the person’s conversation” would go a long way to ensure compliance. For “talking without sound”, this again would be easy to confirm with an analysis of video recording, or if you wanted to get precise, facial EMG. I am not suggesting you deploy fEMG, but this would provide clarity that motor movements between chewing gum and sucking on a lollipop differed in the way that you theorised. Again, a video analysis with a human coder would be the most straightforward solution. For “talking inside the head”, you may wish to consult the imagery literature to identify a suitable reliability task.

Thank you for pointing out the possible problem of compliance concerning our non-verbalization tasks. In experiment 2, we therefore included reliability checks for the cognitive aspects:

- an objective criterion for performance, i.e. the report of the derived result of mental arithmetic, which made it possible to compare the performance during “normal talking” and “talking inside the head”
- a subjective rating of the attentional involvement in the task. Trials with low ratings were excluded form analysis.

In addition, in the “listening” condition participants were asked to monitor the auditory input and report at the end of the trial which number was left out in a stream of easy calculations.

To check for compliance with the motor task, we recorded facial EMG (muscular activity) in both follow-up experiments.

We agree that a video analysis could also have solved some of these issues, however, we decided against the use of videos, because being videotaped might change the behavior of the participants including blink behavior (especially when talking about a personal topic).

I shared reviewer 2’s concerns over the reported blink analysis technique. I am not an expert in eye blink analysis, and will defer to their expertise here, and they seem relatively satisfied. However, it needs to be clarified if this is a novel technique. If so, why was a new eye blink technique required? This is a relatively mature field, and there appear to be more robust eye blink detection methods out there. Why was a dimly lit room used with this optic/vision-based hardware? If this is a novel analysis technique, I would like to see some basic reliability data comparing tracking glass-derived blinks against that of a human coder, or that of a validated technique. This is not a methods paper, and so the sample would not need to be large, but sufficient to give confidence in the technique.

Indeed, blinks have been analyzed for a long time and consequently, reliable detection algorithms are available. A reason for us to develop our own algorithm was to have full control over all parameters (which is not the case when applying commercial software as the one provided from eyetracking companies) and to understand the relationship between the eyes (left/right) or other variables such as gaze. Indeed, a blink as simple as it seems at first, has rather complex features, which should be taken into consideration when trying to understand blink behavior. To name a few:

- not all blinks are binocular;
- there are very short blinks and eye lid flutter;
- there are partial blinks were one or both eyes are not completely shut (some subjects mostly blink like this and a normal algorithm would detect no blinks)

While this information can be very important for researches trying to understand blinking behavior, in the manuscript at hands, we applied our blink detection algorithm in a conservative fashion to secure comparability to previous work.

This comparability was proven by comparing blink detection using our algorithm with other approaches as reported in the supplementary material of our 2021 publication: Brych, M., Händel, B. F., Riechelmann, E., Pieczykolan, A., & Huestegge, L. (2021). Effects of vocal demands on pupil dilation. *Psychophysiology*, e13729. It includes a comparison of our blink detection algorithm with the internal EyeLink blink detection and an EOG detection algorithm by an independent research group (Wascher et al. (2015) Eye-blinks in choice response tasks uncover hidden aspects of information processing. EXCLI journal. 2015;14:1207.) revealing only very small differences in detected blink rate and duration.

Please note that we used an EyeLink eyetracker in the before mentioned study (Brych et al., 2021), but in the study at hands we used SMI eye tracking glasses for the original experiment (experiment 1). However, as both eye trackers are video based systems, there is no reason to suspect that the algorithm should not work comparably. Additionally, for the follow-up experiments, we added EOG and used the EOG blink detection algorithm described by Wascher et al. (2015) to further prove the reliability of our blink detection algorithm (please see the corresponding methods sections of experiment 2 and 3).

Concerning the illumination during the original experiment, previous studies in our lab showed that the SMI eyetracker recorded data most reliably in the absence of strong light sources (such as sun light, but also artificial lights from certain directions). In addition, we wanted to minimize unnecessary visual input during the experiment. This is why we used a dimly lit room in experiment 1.

The statistics were a strength of the manuscript; however, several issues should be addressed. First, was a prospective power analysis conducted? If not, the implications need to be discussed. In particular, that several conditions in post-hoc tests approached significance, but may not have due to insufficient power.

Concerning experiment 1, no power analyses was conducted. The sample size of our follow-up experiments (2&3) were calculated based on the motor component analysis of the first experiment achieving a power over 95%. Overall, we achieved a high replicability of our results from experiment 1.

We are not completely sure which post-hoc tests approaching significance the reviewer refers to, but assume it is the one comparing “listen to someone else” and “baseline3” (p = .089), as this is the only one with p below 0.1. As neither the Bayesian approach suggests a clear trend nor do we find an effect auditory input in experiment 2, we do not assume to have missed a strong influence on blink rate.

Second, were assumptions of normality etc. assessed prior to running ANOVAs? Like Reviewer 2, the description of ANOVA structure could be made clearer to the reader. For example, “a two-way repeated measures ANOVA was conducted on DV, with X (2 levels: A, B), and Y (3 levels: C, D, E) as within-subjects factors”. Relatedly, the description of the 8 conditions could be improved; I had difficulty tracking them all. There appeared to be a natural grouping, which you may find helpful for improving clarity: baseline (1), speech (3 levels: inside head, movement without sound, normal), motor (2 levels: lollipop, gum), listening (2 levels: someone else, self-recorded). Please also report the software used to conduct statistical analyses, along with associated packages.

Concerning the normality assumption, ANOVAs are highly robust against a violation of normality when equal sample sizes are given. Therefore, we do not explicitly mention this. Concerning sphericity violations, we added a short sentence in the data analysis section:

(manuscript with tracked changes, lines 144-145) “Repeated measures ANOVAs and corresponding post hoc analyses for blink rate were computed. The epsilon for Huynh-Feldt correction is given in case of violation of sphericity.”

We tried to improve our result section to make our analysis steps easier to read. Additionally, we included tables for each experiment that describe the tasks and (if appropriate) mark the use in the different analyses. Used software is reported in lines 146-147.

Reviewer #1

The present study examined which factor induced an increase in blink rate during speaking by comparing blink rate between various conditions. They revealed that the motor activity during speaking was a major factor to increase blink rate. They carefully designed the research and the data analysis using Bayesian estimation was performed properly.

The main claim of the present study is that neither the cognitive processes nor the auditory input, but rather, the motor activity of the mouth has the main influence on our blink rate. They conclude this statement depending on the finding that the blink rate during speaking was significantly higher than that during speaking inside the head. However, I cannot agree to their conclusion because it is quite difficult to assess whether participants will seriously generate the sentence during speaking inside the head. There is a high possibility that the cognitive load between those two conditions were different. Large amount of previous studies consistently reported that cognitive load and arousal significantly influence on blink rate. The present study contradicts with those findings. Further experiment is necessary to support the current findings. For example, an experiment examines whether the blink rate changes when the cognitive load is changed due to the difficulty level of the topic under the same conditions for the motor activity of speaking.

We would like to thank the reviewer for his/her careful reading. We agree that our experiment was not fully able to control the cognitive load especially during “speaking inside the head”. Based on the reviewer’s suggestion, we conducted two follow-up experiments. Experiment 2 was specifically designed to control cognitive load during both “normal talking” and “talking inside the head” including different levels of difficulty. We asked participants to subjectively rate their concentration on a scale from 1 (low) to 7 (high) and excluded all trials in which the rate was less or equal than 3. In addition, participants had to report the last number of their mental arithmetic task in the “talking inside the head” task, which we used to assess the performance in comparison to the “normal talking” condition. Please note, that our results are in line with previous research that the blink rate differs between the easy and difficult conditions, but we could also show that this influence is only weak (as compared to the motor influence) and cannot account for the increase in blink rate during speaking.

Reviewer #2

This is a very interesting, concisely written and important study on the relationship between blink rate and cognitive and motor factors during speech. The idea is very clear: blink rate is increased during speaking, but is this due to the movements involved in speaking or the cognitive effort required of the speaking act. The present study is an elegant one that tries to de-confound the motor and cognitive functions and examines the results in blink rate. I have a fair few comments related to the clarity and completeness of the introduction and methods, but I find the experiment sound, the analysis valid, and the conclusion reasonable. Indeed, I believe this type of work that seeks to test the validity of ‘biomarkers’ of critical importance for the long-term health of science and would like to see the importance of the contribution underlined. To that end, I sincerely you find my comments informative and useful.

We would like to thank the reviewer for his careful reading and were happy to work on the improvement of our manuscript based on the comments. Please see our detailed answers below.

1) blink rate (perhaps include a definition) is assumed to be a physiological indicator of cognitive load. I'm not entirely sure what perceptual load could be, perhaps attentional is meant. Some people have suggested that blink rate is a correlate for dopaminergic activity (c.f. Colzato et al., 2008, Neuropsychologia), which might explain the relation with clinical diagnosis. This will also tie neatly into some of the existing controversies in the cognitive load – blink rate correlation as some failures to replicate have been reported (Sescousse et al., 2018, European Journal of Neuroscience; Dang et al., 2017, eNeuro; although Van Slooten et al., 2019, Psychopharmacology).

Thanks for pointing this out. We give an example of what we mean by ‘blink rate’ at the beginning of our introduction excluding any possible uncertainties. In addition, we agree that the term ‘perceptual load’ is not clear-cut, which is why we rephrased this part. The introduction starts now with the following sentences:

(manuscript with tracked changes, lines 36-40) “Humans blink approximately every 3-6 seconds which is far more than needed to keep a constant tear film on the cornea [1]. During visually demanding tasks such as reading, the blink rate drops from approximately 17 blinks per minute during rest to approximately 4.5. On the contrary, the blink rate increases during conversation to approximately 26 blinks per minute [2].”

The controversy in the relation between blink rate and dopamine-related cognitive function is indeed widely spread, however, given that we do not find a strong influence of cognitive load on blink rate, we don’t describe this discussion in great detail in our paper. Nevertheless we included a short text passage referring to a detailed review about this relation (Jongkees & Colzato, 2016) as well as including your suggested references about the replication failures:

(lines 54-58) “The causes for these deviations from normal blink behavior was long suggested to be connected to dopaminergic hypo- or hyperactivity [10], but more recent studies sow doubts showing no positive relationship between spontaneous blink rate and dopamine synthesis capacity or D2 receptor availability [11, 12].”

2) I would suggest slight restructuring of the first paragraph to first explain blink rate, what it does and why it would involve cognitive load and so on, and then to move the correlation between blink rate and speaking to a second paragraph - currently the second sentence in paragraph 1 seems like a strange shift of focus ("Also, the often found increase in blink rate during conversation").

We hope that the restructured introduction has improved in coherency and readability.

3) Procedure: What does 'no task' mean in the baseline? Is this like a resting state recording in an fMRI experiment?

Yes, we are happy to clarify this in the methods section: “During the baseline conditions, participants should not stand up or close their eyes, but had no additional task, which will be referred to as ‘resting’ ” (lines 126-128).

4) Procedure: Were participants told to come up with topics for themselves or was some randomisation of task x topic done? Perhaps it would help the reader if you could give a table with each of the 8 conditions with a short explanation.

The topics (e.g. “What are your hobbies”, “How do you cook spaghetti with tomato sauce, “What do you like / do not like about your university”, “Describe your home town”, …) were defined by us and topics were randomized across all tasks and participants. We added the following information in the text:

(lines 119-120) “Topics were defined by us and randomized across tasks and participants.”

Each experimental description now includes a table giving an overview and short descriptions of the tasks.

5) Procedure: I imagine participants didn't immediately know what to say during talking conditions, was the length of the task 1 minute from onset of talking or onset of instruction?

We apologize that this information was omitted in the original draft. After having heard the instruction, participants would start the 1 minute trial by pressing a button whenever they felt ready. Subjects therefore had sufficient time to think about the topic before they started the trial, but few made extensive use of this possibility given that the topics were fairly easy to talk about. We edited the corresponding sentence in the methods to better describe the paradigm:

(line 131) “Participants were able to start each trial at their own pace by pressing a button followed by a starting tone. The end of the trial was signaled by another tone.”

6) Data analysis: Digitally recorded speech can be represented as waveforms, it's strictly speaking not transformed. Also, I do not understand what 'which were controlled for outburst signalling continuous talking' means. What is controlled? Is an 'outburst' a sudden high intensity or do you just mean if there's any amplitude in the waveforms it suggested continuous talking? Or is a noise gate filter applied?

We are sorry for the confusion. We did not listen to the speech, since we did not get the participants’ approval to listen to their monologues. Any amplitude that was notably above baseline served as control for task fulfillment. The sentence was rephrased:

(lines 140-141) “We visually inspected the waveforms representing speech to control for task fulfillment in the ‘normal talking’ condition.”

7) Blink detection: This is to some extent part of data analysis and might be better presented as part of that.

We had given the description of blink detection approach an extra section in order to facilitate reading and highlight this important information. If the reviewer insists, we can of course integrate it into the data analysis section.

8) Blink detection: I have some problems with identifying the blink with the pupil radius. That is, I understand that you get pupil radius values from the SMI recordings, but 1) pupil radius tends to be very strongly affected by whatever people are looking at, and 2) if people close their eyes, their pupils increase in size (due to lower light intensity reaching the eye through the lid), not decrease. Perhaps if SMI doesn't see a pupil, or the pupil is partially occluded, it outputs values of them that are small, but that seems an idiosyncracy of the equipment. That said, I find the detection clear enough, it's just that the biological (pupil size, blink) should not be confused for the technological. Please rewrite the preprocessing steps with this in mind.

The reviewer is without doubt correct in his description of pupil size and blinking. As he further suggests, SMI eye tracking glasses, as video-based eyetracker, record the size of the pupil as long as it can be detected. When the eyelid covers the pupil during blinking, the recorded pupil size quickly reduces and then is marked as “not-a-number” as the pupil is undetectable. We take this, as the reviewer puts it, “idiosyncracy of the equipment” as indicator of a blink. This signal change is indeed distinct from a real change in pupil size as introduced by blinking, cognition or sensory input. We hope to have made this clear in the updated description, which now read as:

(lines 149-153) “When the eyelid occludes the pupil during a blink, pupil size recordings of video-based eye tracker quickly and strongly decrease until the pupil is undetectable. Using this characteristic, our blink detection algorithm is based on the recorded pupil size. Blinks were initially detected when both z-transformed pupil radii were below a threshold of -2 standard deviations or when the pupil data was marked as lost.”

Please note that the supplementary material of our previous publication (Brych, Händel, Riechelmann, Pieczykolan, Huestegge (2021) Effects of vocal demands on pupil dilation. Psychophysiology. 58(2):e13729) includes a comparison of our blink detection algorithm with the provided blink detection by EyeLink and Wascher’s EOG blink detection (used for experiments 2&3).

9) Results: In general, I would advice a little bit of preamble and restructuring. Currently, the analyses seem to drop one after another and it is not always clear what hypotheses you are exactly trying to prove/disprove. Am I correct you are first trying to estimate the effect of talking on blink rate, the basic effect of interest; then to see whether this effect is best approximated by lip movements rather than chewing movements; and then by listening? Both the introductions and method sections could more easily provide predictions, and overview of analysis.

Yes, we analyzed the cognitive influence first, followed by the motor components and finally auditory influences. We added this information in the tables describing the different tasks and improved the result section.

10) Results: The second factor ('repetition') makes very little sense if there is no clear manipulation. If the randomisation was by condition first and repetition second (repeating each condition once before re-randomising) rather than full randomisation, then the repetition suggests a factor of time, which could of course have an effect, but this is not explained.

We agree that the factor ‘repetition’ is not needed and changed our analysis by averaging over the repetitions before comparing the different tasks. The sentence about our randomization was improved to make sure readers understand that the timing within the experiment cannot affect blink rate for any specific condition:

(lines 128-129) “The order of tasks was fully randomized to exclude any time related effects […]”

11) Results, classic analysis: I am not sure which factor is used in the 2-way repeated measures analysis: is it between the 3 conditions, between the 8, or between the 3 and then the 5 remaining? Please use a standard type phrase ('A repeated measures ANOVA with Condition (baseline vs normal talking vs talking inside the head) and Repetition (1st to 5th) as factors - or something to that tune, then write out the analysis. I think the rest of the analysis concerns the differences between the 3 conditions, but what happened with the other 5?

As stated above, we edited the result section similar to what you suggested. The tables for task description as well as the figures should additionally reduce uncertainties.

12) Results, Bayesian: A Bayesian analysis is not done to assess the magnitude of differences (that's the effect size), but rather to estimate the evidence of the hypothesis given the evidence. However, I am not entirely sure what the null- and alternative hypothesis are - please specify. It would make things a bit easier if the repetition factor were entirely eliminated from the analysis (as the null-model can both include and not include it currently). Instead, if you are interested in revealing magnitude of differences and the partial eta squares provided are not enough (they rarely are), I would appreciate a simple magnitude (e.g. normal talking increased blink rate by ca. 100%).

Thank you, we highly appreciate this comment. We eliminated the factor “repetition” and improved the paragraphs of Bayesian analysis according to your information.

JASP uses a “null control” correction for post-hoc tests as a default, which is what we currently applied in our analysis. We would be interested to learn if you think this is not the correct use of Bayesian post-hoc tests for our experiment.

13) Discussion: The conclusions seem sound and follow the data analysis. I was a bit disappointed that the authors did not follow up their analysis with an exploratory analysis of the number of syllables uttered and blink rate, as that seems not too prohibitive an amount of additional work. Perhaps that might be added as supplementary analysis?

Thank you. Unfortunately, we did not get the permission of the participants to listen to their speech in experiment 1. We therefore could not analyze the number of syllables uttered. However, we followed up on this idea in experiment 2, but did not find any influence. However, this might be due to the fact that no full sentences were spoken but merely numbers were uttered.

14) Discussion: I very much agree with the suggestion that blink rate should not be seen as somehow a pure indicator of cognitive load. As to that, I would recommend the authors to phrase their caution even stronger, and not merely in the context of patient interviews, but also experimental work. It is quite possible, for example, that cognitive load or task difficulty could lead to affective responses expresed by the mouth, which may then lead to measurements of blink-rate patterns that are only indirectly related to the suggested causal mechanism (c.f. Maffei & Angrilli, Physiology and behavior, 2019). In that sense, the present study had a very clear focus on speech, but the results may well transfer to other domains, and the finding that blink rate does not provide a process-pure measure of cognition (or emotion, for that matter) is a critical one to get across.

Yes, our results show that cognitive load has only a minor influence on blink rate. Also, our follow-up experiments (2&3) specified that complex motor activity, but not isolated mouth movements, act as a driving factor of blink rate increase. Therefore, small movements e.g. during emotionally driven facial expressions are rather unlikely to influence blink rate substantially, however this could be investigated further.

Following your suggestion, we extended our recommendation beyond patient settings:

(lines 669-670) “Given our results, we advise caution when using blinks as neurological indicators during patient interviews or as indicators of cognitive load during tasks involving verbal responses.”
