# Supplementary material for: How the motor aspect of speaking influences the blink rate
Source: PLoS One. 2021 Oct 8;16(10):e0258322. doi: 10.1371/journal.pone.0258322 (PMC8500445; doi:10.1371/journal.pone.0258322)
Supplement: Attachment — Submitted filename: ResponseToReviewers_minorRevision.docx [file pone.0258322.s002.docx]

**To both reviewer: We would like to thank the reviewers for their careful reading and are happy to improve our manuscript based on the suggested comments. Please see our detailed answers below.** Line references refer to the file manuscript_trackedChanges.docx

Please note that we found a mistake in the analysis program of experiment 2 (lines 433-510). The results slightly change, but did not influence our line of argumentation. We are sorry for the mistake in the original version.

In addition to our data files, we also uploaded our MATLAB scripts on the public repository OSF.

Reviewer #2: Thank you for one of the most solid responses to reviewers I have ever had the pleasure to receive. I'm impressed by the changes (including additional experiments), the completeness of the response letter, and the final manuscript. No further comments!

Thank you very much. We appreciate your comment!

Reviewer #3: Comments

Introduction

The research aim seems to be clearly stated in L64-L66. However, I find it difficult to extract a clear rationale for the research in general. Why is it interesting to find a relationship between blinking behavior and isolated muscle movement and potential concurrent cognitive effort? The authors state in L41-L44: “A clarification of the influence of motor activity seems relevant, especially since blinks serve as neurological indicators in clinical settings. For example, Parkinson’s disease is associated with very low blink rates [8], while high blink rates are observed in patients with Schizophrenia [9].”. It is however not clear to me how the relationships between blinking rate and the different tested conditions could be clinically related to e.g., the aforementioned illnesses. Please provide a clearer rationale. If blink rate is not mediated by dopaminergic hypo- or hyperactivity, and – as suggested by the authors – hypothetically through “facial motor activity”, then the authors should cite plenty sources as to the origins of this hypothesis. See also the next comment.

Thank you for pointing this out. We added a clear rational in the end of the general introduction (lines 74-77): “*Apart from the new insights on how blinks and other body movements are related, our work seeks to clarify the validity of blinks as a marker for pathological states as well as for sensory and cognitive processing in experiments using verbal responses.*”

We are sorry for the unclear phrasing and improved our introduction part on the potential clinical relation. It now reads as:

*“For example, very low blink rates are observed in patients with Parkinson’s disease [11], which is possibly due to dopaminergic hypoactivity [review by 12, questioned by 13, 14]. The patient’s response to medication can be assessed by the increase in blink rate, which is often measured during the conversation with the physician [11]. Consequently, if other factors such as speaking increases blink rate in the same direction, this might lead to inaccurate medical examinations.”* (lines 44-50).

Please not that we are not arguing that the dopaminergic activity is irrelevant and the motor activity is most relevant, but that both can have an influence on our blink behavior.

In L67-L75 the authors continue to provide some hypotheses, however I find no references to literature that give rise to such hypotheses in the first place. Please cite relevant sources that back your claims.

Yes, thank you. We added several references. Please see lines 88-98.

Also, specifically in L71-L75 the authors already discuss the results from the first experiment. I believe they have done this they appended the paper with further experiments, However, it is no good practice to discuss results already in the introduction, so please omit this section from the introduction.

We followed your suggestion and deleted the paragraphs referring to results from experiment 1 from the introduction.

Methods exp.1

Please include a visual schematic as to how the subjects were seated relative to the experimental equipment. (if identical for all three experiments, one schematic would suffice)

We added a schematic of experimental equipment which was except for experiment 2 mainly attached to the participant (figure 1).

The eight different experimental conditions, e.g. “talking inside the head” and “lollipop” seem not to be sufficiently motivated. Why have the authors choses specifically these activities and could they cite other sources on the credibility of these conditions.

We agree that we haven’t fully laid out our reasons to use these conditions. Therefore, we added some information (starting in line 116):

*“When measuring blink rate during a conversation, there are several possible influences. Our different experimental conditions were designed to test for influences of cognitive load during speech production (with and without vocalization), of motor output (mouth movements, with focus on lip or jaw movements) and of auditory input (due to one’s own speaking or someone else). “*

Concerning “talking inside the head”, see line 127-129: “*Talking inside the head involved no mouth movement and no sound production, but required cognitive processes that are comparable to the cognitive processes during normal talking*”. During the first round of this manuscript, our old editor/our reviewers noted that there is no reliability whether participants actually performed the task. Consequently, we changed the cognitive tasks in experiment 2 into mental arithmetic, which made it possible to obtain some performance measurement.

Gum chewing inducing jaw movements was previously used (we refer to the study by Karson et al. in the introduction, see line 39).

Sucking on a lollipop to induce lip movements was indeed not used previously by other researchers. There are several reasons why we chose this movement. First, it is fairly easy to perform this task throughout a whole minute (or even longer, if by chance this condition came in succession). Second, our participants were naïve to our actual experimental purpose. Third, not many common actions require constant lip movements (we rejected whistling, because not everyone is capable of doing so, and rejected kissing moves due to the possibility of high emotional load). Please note, that throughout the manuscript, we come to the conclusion that the “lollipop” condition likely involves more complex movements than just lip movements, which we tested in experiment 2.

We added on line 133-136: “*We chose sucking on a lollipop as easy way to induce mouth and especially lip movements. Respectively, gum chewing intended to mainly introduce jaw movements. However we are aware that also other movements such as tongue movements and swallowing are likely executed as well*.”

“*Talking without sound was used to mimic mouth movements as during normal speaking but omitting auditory stimulation”* (we added this information in line 130-131).

For the listening conditions, we also added some information. See line 139-141: “*Listen to oneself therefore is the same auditory input as during the normal talking condition, however, “listen to someone” else was added to mimic the auditory input experienced during a conversation with another person*.”

Overall, our choices were made to control for various possible influences of speaking on blink rate that might contribute individually or in combination to the overall effects of the blink rate increase. We do not cite many sources, because we are not aware of any study that controlled for all the potential sources of speech-related influences.

What does “Additionally, we visually inspected the waveforms representing speech to control for task fulfillment in the “normal talking” condition.” Mean?

We are sorry for the confusion. We tried to improve the sentence by adding additional information. See line 157-160 “*Since we did not have the participant’s permission to listen to the monologues, we plotted the recorded sound signal and visually inspected the amplitude of the signal representing speech to control for task fulfillment in the normal talking condition*.”

Could the authors provide us with a pseudocode of the novel blink detection algorithm? And also discuss how it differs from other state-of-the-art methods?

Indeed, blinks have been analyzed for a long time and consequently, reliable detection algorithms are available. However, eyetracking companies (SMI and Eyelink) do not make their blink detection algorithms publicly available. A reason for us to develop our own algorithm was to have full control over all parameters and to understand the relationship between the eyes (left/right) or other variables such as gaze. Indeed, a blink as simple as it seems at first, has rather complex features, which should be taken into consideration when trying to understand blink behavior. To name a few:

- not all blinks are binocular;
- there are very short blinks and eye lid flutter;
- there are partial blinks were one or both eyes are not completely shut (some subjects mostly blink like this and a normal algorithm would detect no blinks)

While this information can be very important for researches trying to understand blinking behavior, in the manuscript at hands, we applied our blink detection algorithm in a conservative fashion to secure comparability to previous work.

This comparability was proven by comparing blink detection using our algorithm with other approaches as reported in the supplementary material of our 2021 publication: Brych, M., Händel, B. F., Riechelmann, E., Pieczykolan, A., & Huestegge, L. (2021). Effects of vocal demands on pupil dilation. *Psychophysiology*, e13729. It includes a comparison of our blink detection algorithm with the internal EyeLink blink detection and the EOG detection algorithm by an independent research group (Wascher et al. (2015) Eye-blinks in choice response tasks uncover hidden aspects of information processing. EXCLI journal. 2015;14:1207.) revealing only very small differences in blink rate and duration.

Please note that we used an EyeLink eyetracker in the before mentioned study (Brych et al., 2021), but in the study at hands we used SMI eye tracking glasses for the original experiment (experiment 1). However, as both eye trackers are video based systems, there is no reason to suspect that the algorithm should not work comparably. Additionally, for the follow-up experiments, we added EOG and used the EOG blink detection algorithm described by Wascher et al. (2015) to further prove the reliability of our blink detection algorithm (please see the corresponding methods sections of experiment 2 and 3).

**Pseudocode** of video-based blink detection:

Get pupil data of both eyes

Calculate z-values of pupil size for each eye separately

Mark where pupil size is below manual threshold (-2 SD) or undetectable for each eye

Only mark periods when both eyes are below threshold/undetectable (no monocular blinks)

Extend marked periods until one eye’s pupil size reaches half the threshold

Combine temporally very close blinks (e.g. lid flutter)

Delete blinks that are very long (e.g. sleeping)

Results exp.1
The authors should elaborate more on the added value of Bayesian analysis in comparison to the (well executed) regular frequentist statistics.

Frequentist statistics are well suited to determine statistical significance in an all-or-none fashion. We added Bayesian analysis as a supplement in a way that we can acquire knowledge on the credibility of the alternative hypothesis as well as of the null hypothesis. We added the following sentence in the data analysis section of experiment 1 (lines 162-164): “*Bayesian analysis was added as a supplement to the classical frequentist statistics to get insights on the credibility of the alternative as well as the null hypothesis*”.

Discussion exp.1
From L274 onwards, the aim of the second experiment is explained, however it feels more appropriate to include this text in a small introduction section - similar to L29-L75 – rather than putting it in the introduction section of experiment 1.

Yes, we agree. We transformed the last part of the discussion into an introduction as you suggested. (lines 329-337)

Experiment 2:

Blink detection: are eye-tracker and EOG data comparable in temporal resolution with respect to blink detection? Could the authors include a metric that quantifies the overlap between the two methods?

The SMI eyetracker has a sampling rate of 120 Hz, the EMG (including EOG) 500Hz, the Eyelink eyetracker 500 Hz (we added this information in the methods sections). There were several reasons why we could not directly compare blink on- and offsets between methods. First, the difference in signals makes it very hard to find the exact overlap. Second, we found a minimal, changing offset between time streams. However, our results are based on blink rate (number of blinks in a minute), not blink timing and are therefore suited for our analyses. The blink rates between methods are highly comparable (for Eyelink vs custom blink detection vs EOG, see supplementary material of our previous publication: Brych, M., Händel, B. F., Riechelmann, E., Pieczykolan, A., & Huestegge, L. (2021). Effects of vocal demands on pupil dilation. *Psychophysiology*, e13729). Please note that we chose to record eye movements with several methods to be able to use the most accurate recording among them given that each method has advantages and disadvantages such as high/low light sensitivity or high/low robustness against movements.

It is not clear to me how performance on the mental arithmetic conditions between (and within) participants differed. What was the general level of performance of the participants?

In order to facilitate this result section, we included a figure for performance (fig. 4a). In the easy task, participants made more than 50 calculations, during the difficult task around 20 calculations.

Is there any correlation between task performance and blink rate?

This is indeed a very interesting question; however unfortunately, our data seems not quite suited to answer this question. If we test for within participants’ effects, we would need to test for a correlation based on 5 data points (5*1 minute), which is little to extract reliable relationships.

When assessing a between subjects analysis, i.e. if performance varies between low or high blinkers, no pattern was evident.

Also: for me it is not entirely clear how the authors defined an “error” in case of ‘arithmetic in the head’. E.g. if in a minute a subject subtracts 7, 14 times from 200. Ideally the subject would arrive at 102. However, what if the subject reported 100? Or 104? How did the authors infer a performance metric from these ‘singular’ reports?

We decided to recalculate the performance based on the number of maximal possible calculations. The results did not change. To make it easier to understand, we also added an example. The manuscript was changed in the following way (lines 405-424):

“*To evaluate the task demands on performance, we took the entered last number during “calculating inside the head” conditions and extracted the last number of the recorded speech during “calculating aloud” conditions to quantify calculations in the same way for all conditions.”* Thus, we chose singular reports to make it more comparable between conditions. It further says:

*“If the last number was not a number obtained after correct calculation (only difficult conditions), we counted calculations that were possible up to this point. For example, after 14 calculations, the participant should have arrived at 102, but entered 105. Then, 13 correct calculations were possible and minimally one error. In case of 100, 14 correct calculations were possible. Please note, that this quantification could only result in an overestimation of correct calculations in case of an incorrect last number, and thus, was rather conservative as it made the analysis less likely to find a difference between easy and difficult mental arithmetic. The error identification in the “calculating aloud – difficult” condition, where participants made approximately one error per trial (mean: 1.32, SD: 1.11), supported our approach to only assume one error if the last number was incorrect. As for the blink rate analysis, trials were excluded after which participants evaluated their performance less or equal than 3 on a scale from 1 to 7 (i.e. one participant was excluded for this analysis).”*

Following your example, 100 would result in 14 possible correct calculations and minimally 1 error (the error of subtracting 2 instead of 7). 104 would result in 13 possible correct calculations and minimally 1 error (the error of subtracting 5 instead of 7). We decided not to differentiate errors, such as subtracting 2 might be more unlikely than subtracting 5, due to the variety of errors, such as subtracting 17, 13, 9, 6, 5, …

Please note, that this attempt is rather conservative in a way that the performance can only be overestimated, which makes it harder to find a difference between easy and difficult conditions.

L485-L491 – same comment as above. It seems that a small, separate introduction seems appropriate

Yes, thank you. We transformed the last part of the discussion into an introduction of the subsequent experiment as you suggested. Each experiment has a short introduction now.

Experiment 3:

Was a prospective power analysis done for experiment 3, if so, could the authors report the results?

We chose the number of participants in experiment 3 upon the results of the power analysis for experiment 2 based on the data of experiment 1. We added this information in the text (see lines 577-579).

L507-L508: was the baseline condition repeated for 15 times, as in the other experiments? Please report on this.

No, 5 minutes of blink rate were recorded. Please note, that also during the other experiments, the baseline conditions included 5 minutes, a duration that was previously suggested by other researchers (Zaman & Doughty 1997 Some methodological issues in the assessment of the spontaneous eyeblink frequency in man). We needed 15 min in total for the other experiments, since we wanted to test each possible influence (cognitive, motor, auditory) with a separate baseline condition in order to avoid adjusting alpha for multiple comparisons. Indeed, this information was missing. It is now added in the methods section of experiment 1 & 2 (lines 143-146 and 382-384).

In general: it was not reported if the participants differed between experiments or if the participants were different. This seems important, as I would expect a “lollipop” condition with experiment 3. Why did the authors not include a “lollipop” condition in experiment 3 for comparison purposes? It seems to me that this was the primary reason for doing experiment 3 in the first place.

We are not sure if we fully understand this comment.

First of all, none of the participants participated in more than one experiment. To clarify this, we added the sentence “*None of the participants took part in the other experiments*” in both methods sections.

Secondly, experiment 3 was not designed to only replicate previous results, but tried to investigate the possibility that the tongue was responsible for the blink rate increase during the lollipop condition as well as during the speaking conditions (aloud and without sound). Given that the tongue alone did not have an effect on the blink rate, we concluded that the combination of lip, tongue and possibly other speech-related movements were responsible for the increase in blink rate during the lollipop and the speaking conditions. In addition, a direct comparison of “normal talking” and “talking without sound” gave further insights on the influence of complex motor activity (as “normal talking” includes mouth movements *and* vocalization, but “talking without sound” only includes mouth movements).

In L562-L563 the authors start talking about pupil diameter and the usage of short syllables. To what extent is this observation valid within the scope of this paper? The introduction of these subjects seems rather ad hoc, and I would advise to omit the sentence.

Following your suggestion, we decided to delete the sentences.

General discussion:

L588-L592: it would be nice if the authors could circle back to the general intro, by explaining how the current results are related to the larger picture they paint – the clinical applicability of blinking as a neurological indicator. Since the authors did not include a between subject condition in which they tested several groups, e.g., people with Parkinson’s or schizophrenia, it seems that the clinical applicability of the current results are not as evident as they put it here. As said above, this general (‘clinical’) narrative seems not to be backed by the experiments here, and needs to be revised, both in the general introduction, as well as in the general discussion.

We agree that our phrasing put too much emphasis on the clinical applicability. We edited the introduction to make the usefulness of our results for experiments investigating sensory or cognitive influences on blinks under verbal responses more evident and shortened the clinical part. In addition, our aim was to understand movement interaction. We hope to have improved the corresponding parts in both introduction and discussion.
